# Overcoming Vascular Graft Challenges in Tissue Engineering: A Review

**DOI:** 10.3390/bioengineering13101170

**Published:** 2026-10-08

**Authors:** Riya Gandhi, Nora Molten, Selorm Dente, Quint Stein, Angel Artega, Emmett Reid, Narutoshi Hibino

**Affiliations:** Department of Cardiac Surgery, University of Chicago Medicine, S Ellis Ave, Chicago, IL 60637, USA; noramolten@gmail.com (N.M.); sedente@uchicago.edu (S.D.); wstein@uchicago.edu (Q.S.); arteaga@uchicago.edu (A.A.); ereid@uchicago.edu (E.R.); narutoshi.hibino@bsd.uchicago.edu (N.H.)

**Keywords:** vascular grafts, stem cell therapy, cardiothoracic surgery, 3D bioprinting, graft stenosis

## Abstract

The development of patient-specific vascular constructs is essential for advancing clinically effective 3D-bioprinted and tissue-engineered grafts. Optimizing cellular composition and promoting robust vascularization within engineered tissues is important for building an effective vascular construct. In cardiac and vascular patches in particular, insufficient microvascular development often leads to diffusion limitations, ischemia, and eventual necrosis, representing a major barrier to clinical translation. This review examines the challenges of designing constructs that are both anatomically functional and adequately vascularized. We discuss current strategies for tailoring cell ratios and enhancing vascular maturation, as well as emerging technologies in bioprinting, biomaterials, and computational design. Together, these innovations highlight future directions for creating grafts capable of functional integration and long-term survival in patients.

## 1. Introduction

Within reconstructive medicine, a vascular graft is designed to restore blood supply by serving as a viable surrogate for native vessels when the original vasculature is compromised [1,2]. Vascular constructs are most commonly applied as vascular grafts, which are classified by their material origin and dimensions. Autografts are composed of materials from the patient’s own tissue, whereas allografts or alloplasts are derived from a donor or outside cell-based source [3,4]. Although autograft implantation remains the gold standard for transplants, its utility is constrained by lack of available donor vessels.

**Figure 1:**
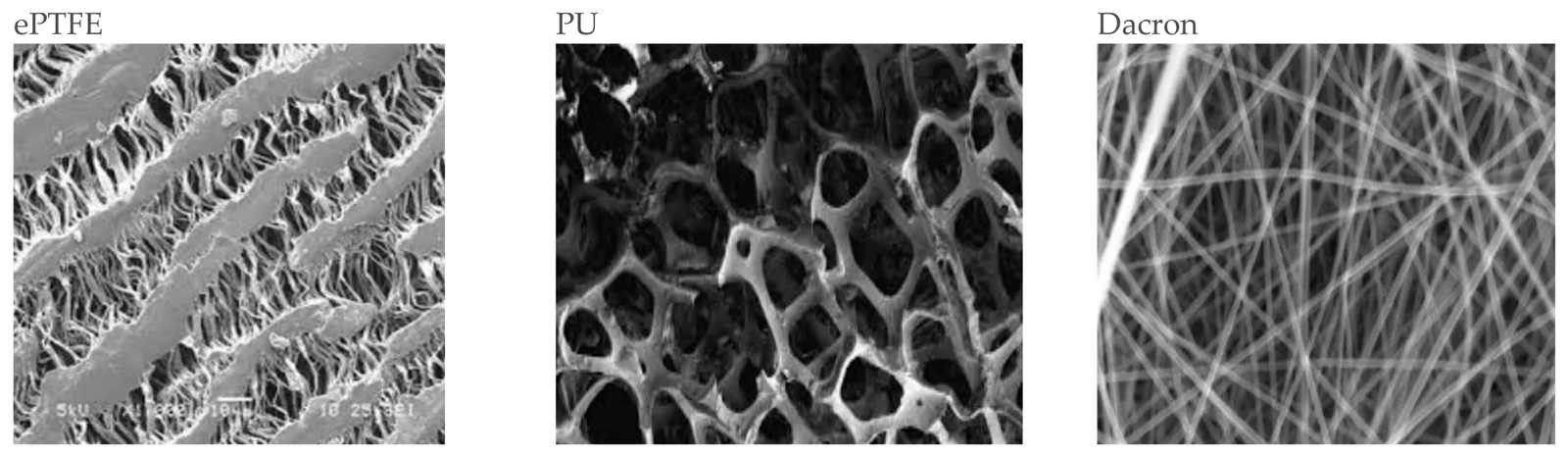
Common materials for allografts: expanded polytetrafluoroethylene (ePTFE), polyurethane (PU) and polyethylene terephthalate (Dacron).

Cardiovascular disease (CVD) is the leading cause of global mortality, accounting for approximately 19.1 million deaths annually, with coronary artery disease (CAD) alone representing 41.2% of these fatalities [5]. In the United States, the prevalence of CAD necessitates roughly 400,000 coronary artery bypass graft (CABG) procedures each year [4,6]. However, a significant clinical bottleneck exists: up to 30% of patients lack suitable autologous donor vessels due to prior surgical harvesting, underlying vascular pathology (such as severe varicosities or atherosclerosis), or anatomical variations that render the vessels unsuitable for grafting [4]. These constraints underscore the urgent clinical requirement for the development of issue-engineered vascular grafts that can provide reliable, non-thrombogenic alternatives to traditional autografts [1,4]. Consequently, much research has focused on allografts constructed from synthetic polymers such as Figure 1 polyurethane (PU), polyethylene terephthalate (Dacron), and expanded polytetrafluoroethylene (ePTFE) [4].

Vascular grafts are also characterized by their internal diameter. Large-diameter vascular grafts (LDVGs) are typically defined as >6 mm or >8 mm, and are typically successfully constructed, whereas small-diameter vascular grafts (SDVGs) or grafts with diameters of less than 6 mm remain a significant challenge [4,7,8]. SDVGs are particularly important because coronary artery reconstruction or bypass surgery (CABG procedures) rely on these grafts, since coronary arteries themselves have a smaller internal diameter, and the inability to produce reliable SDVGs contributes to a 30% deficit in suitable donor tissue for cardiac patients [4,9]. Therefore, development of tissue engineering vascular grafts (TEVG) to solve these challenges is of significant importance. The engineering of vascular grafts involves a complex intersection of mechanical resilience and biological hemocompatibility [10]. Mechanically, vascular constructs must withstand constant pulsatile pressure and cyclic mechanical loading, while cardiac tissues must generate coordinated contractile force and propagate rapid electrical signals [5,10]. Without a functional, non-thrombogenic endothelial lining, blood-contacting surfaces rapidly trigger protein deposition and lumen occlusion [1,5]. We must consider several challenges when constructing a cardiac or vascular graft.

Another approach is creation of micro-scale vasculature (engineered microvasculature) to support small-scale tissue or patches created via stem cells such as mesenchymal stem cells [11,12]. The focus of such engineered vasculature is to allow for better perfusion and angiogenesis during host tissue integration, as well as reliance on stem cells instead of synthetic materials, a scaffold-free approach, to create the grafts, leading to a more stable graft and repair mechanism and better recipient graft tolerance. However, efficient engineered microvascular creation to support thick tissue still faces problems relating to central tissue necrosis due to the 200 um diffusion limit as well as problems with scalability [13,14].

Therefore, this paper will review in depth the current challenges facing the development of vascular grafts and the current solutions and ongoing work to overcome these barriers, as well as future directions for the development of vascular grafts. This paper uniquely focuses on several methods for TEVG development, combining and analyzing points of view from different methods of creating vascular grafts, including scaffold-based microvasculature, and various forms of 3D bioprinting, thus offering a unique perspective on the current landscape of the challenges, limitations and future of vascular grafts.

## 2. Section 1: Challenges of TEVG Development

### 2.1. Fabrication Strategies: Methods and Limitations

#### 2.1.1. Scaffold-Based Approaches to Create TEVGs

**Figure 2:**
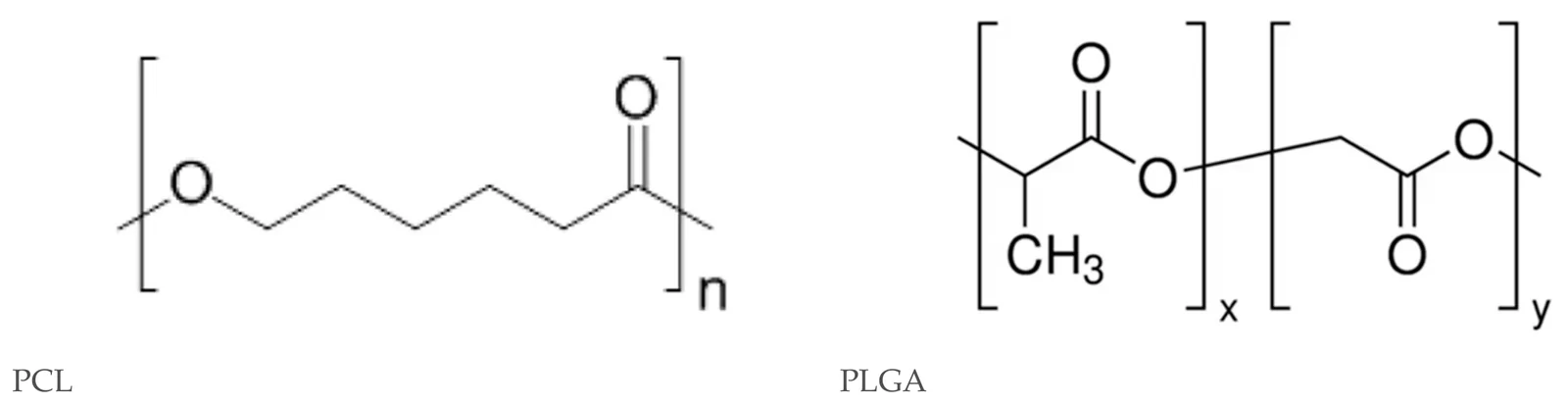
Synthetic polyesters, polycaprolactone (PCL) (**left**) and poly(D,L-lactide-co-glycolide) (PLGA) (**right**).

Scaffold-based biofabrication utilizes three-dimensional biomaterials to provide structural support and guide cellular organization into functional tissues [5,15]. Synthetic polyesters (Figure 2), such as polycaprolactone (PCL) and poly(D,L-lactide-co-glycolide) (PLGA), are frequently employed due to their tunable mechanical properties and FDA approval for specific clinical use [4,5].

**Figure 3:**
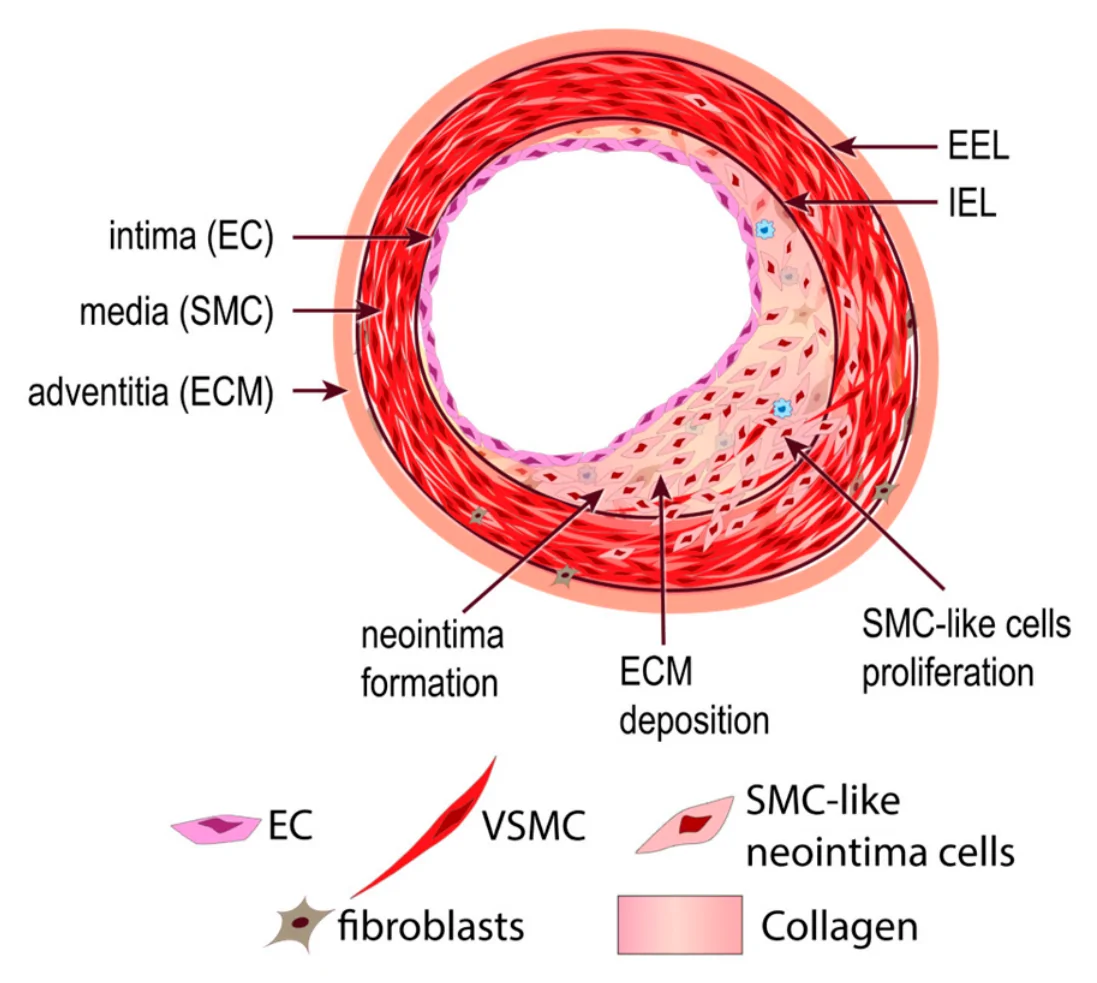
Example of intimal hyperplasia (IH) (Martin, J. 2023) [18].

These materials are often processed via electrospinning to create nanofibrous meshes that replicate the topographical cues and fibrous architecture of the native extracellular matrix (ECM) [5,16]. While these scaffolds offer high mechanical integrity, they often suffer from poor cellular infiltration, with cells primarily populating the surface rather than penetrating the core [5,17]. A central challenge in scaffold engineering is that most conventional synthetic biomaterials are merely “biotolerated” rather than truly biocompatible [4]. Upon implantation, these materials trigger a persistent foreign body reaction (FBR), where the host immune system isolates the construct within a dense, non-vascularized collagenous capsule [1,4]. This fibrotic encapsulation acts as a physical barrier to nutrient exchange and inhibits true integration with the host tissue [4]. Furthermore, this capsule is often constrictive and rigid (Figure 3); in vascular applications, this can narrow the vessel lumen, create significant hemodynamic flow disturbances, and exacerbate intimal hyperplasia (IH) [4,5].

**Figure 4:**
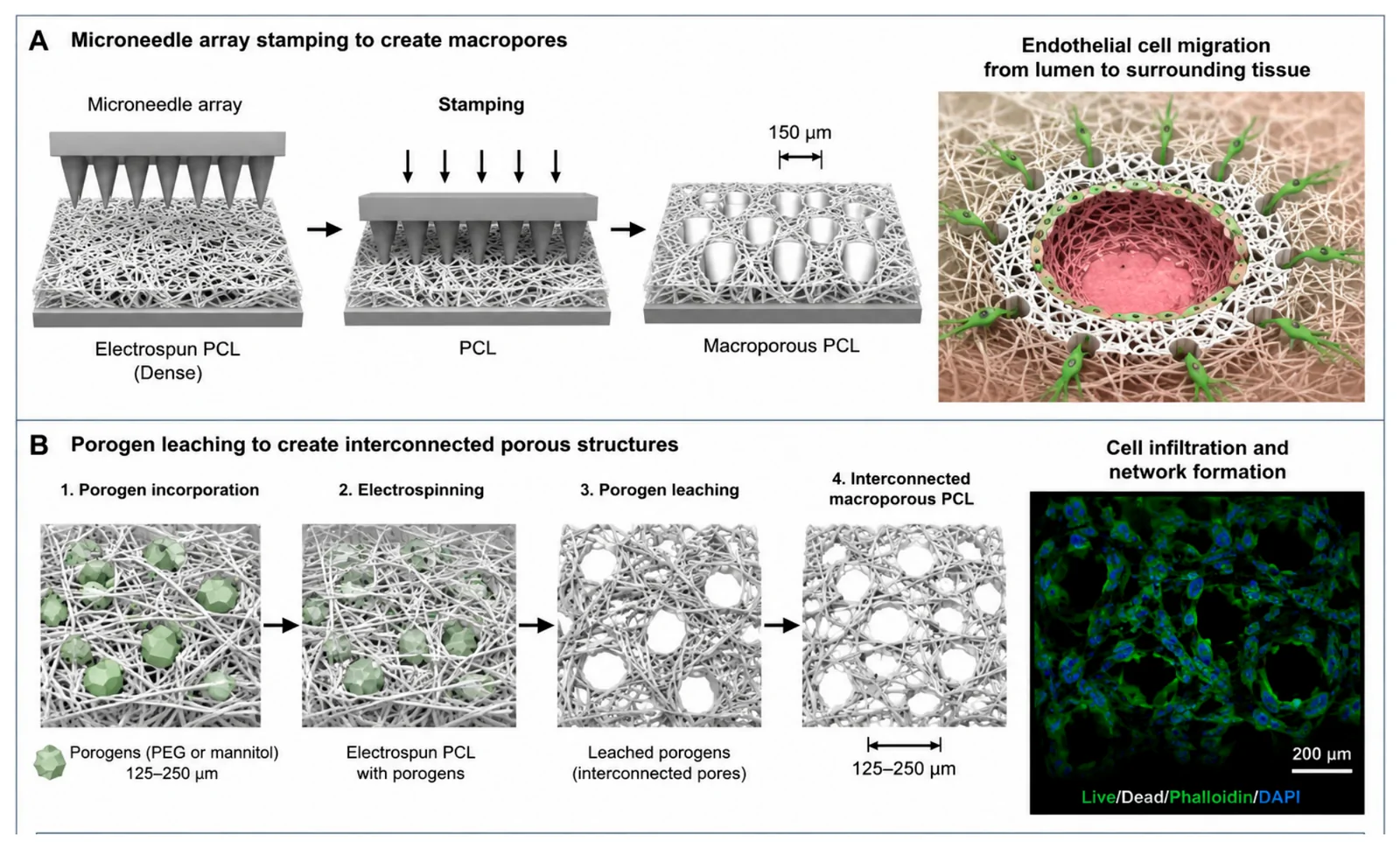
(**A**) Schematic illustration of macropore generation in electrospun PCL scaffolds using microneedle stamping. (**B**) Porogen leaching methods to create porous graft structures. Figure generated with ChatGPT (Version 5 OpenAI) and adapted from Alkazemi et al. (2024) [16].

Mechanical compliance matching is essential; stiffness mismatches between a rigid synthetic graft and elastic native tissue create abnormal hemodynamic patterns that signal smooth muscle cells (SMCs) to proliferate uncontrollably, leading to graft failure [4,5]. Porosity must also be optimized; while small pores provide structural integrity, larger pores (~60–100 μm) are necessary to support sprouting angiogenesis and tissue ingrowth (Figure 4) [4,16].

This is achieved in a variety of ways, including using micro-needle arrays to stamp 150 μm macropores into dense electrospun PCL, enabling endothelial cell migration from the lumen into the surrounding tissue [16]. Selective leaching of porogens like PEG or mannitol crystals (125–250 μm) also creates interconnected porous structures that support cell infiltration [5,19].

#### 2.1.2. Scaffold-Guided In Vivo Self-Assembly

A novel solution to the production cycle challenge is the in vivo self-assembly of tubular tissue, where 3D-printed biodegradable scaffolds are implanted subcutaneously for 14 days to generate host-cell-rich vascular constructs (BTCs) [20]. This strategy shortens the preparation cycle by 80% compared to traditional cell-sheet methods, aligning with the timeframe for elective bypass surgery [20].

#### 2.1.3. Hierarchical Assembly and Biofabrication Strategies for Spheroid-Based Grafts

Spheroids are scaffold-free cellular aggregates that leverage natural self-organization to promote dense cell–cell and cell–ECM interactions [21]. Techniques such as the hanging-drop method or liquid overlay allow cells to form 3D microenvironments that better represent in vivo physiology than 2D cultures [21,22]. These models typically exhibit a spatial hierarchy consisting of an outer proliferating layer, an inner quiescent layer, and a central necrotic core [21]. The primary limitation of spheroids is scalability; constructs exceeding 400–600 μm in diameter invariably suffer from nutrient and oxygen deprivation at the core due to passive diffusion limits [21,22]. Organoids offer even greater complexity through tissue-specific differentiation from hiPSCs but similarly lack a functional vascular compartment, which hinders long-term maturation and physiological relevance [22,23]. Current research addresses this by utilizing superhydrophobic coatings to facilitate high-throughput spheroid formation and recovery [24] or by fusing organoids with pre-vascularized units to establish primitive microvascular networks [23].

**Figure 5:**
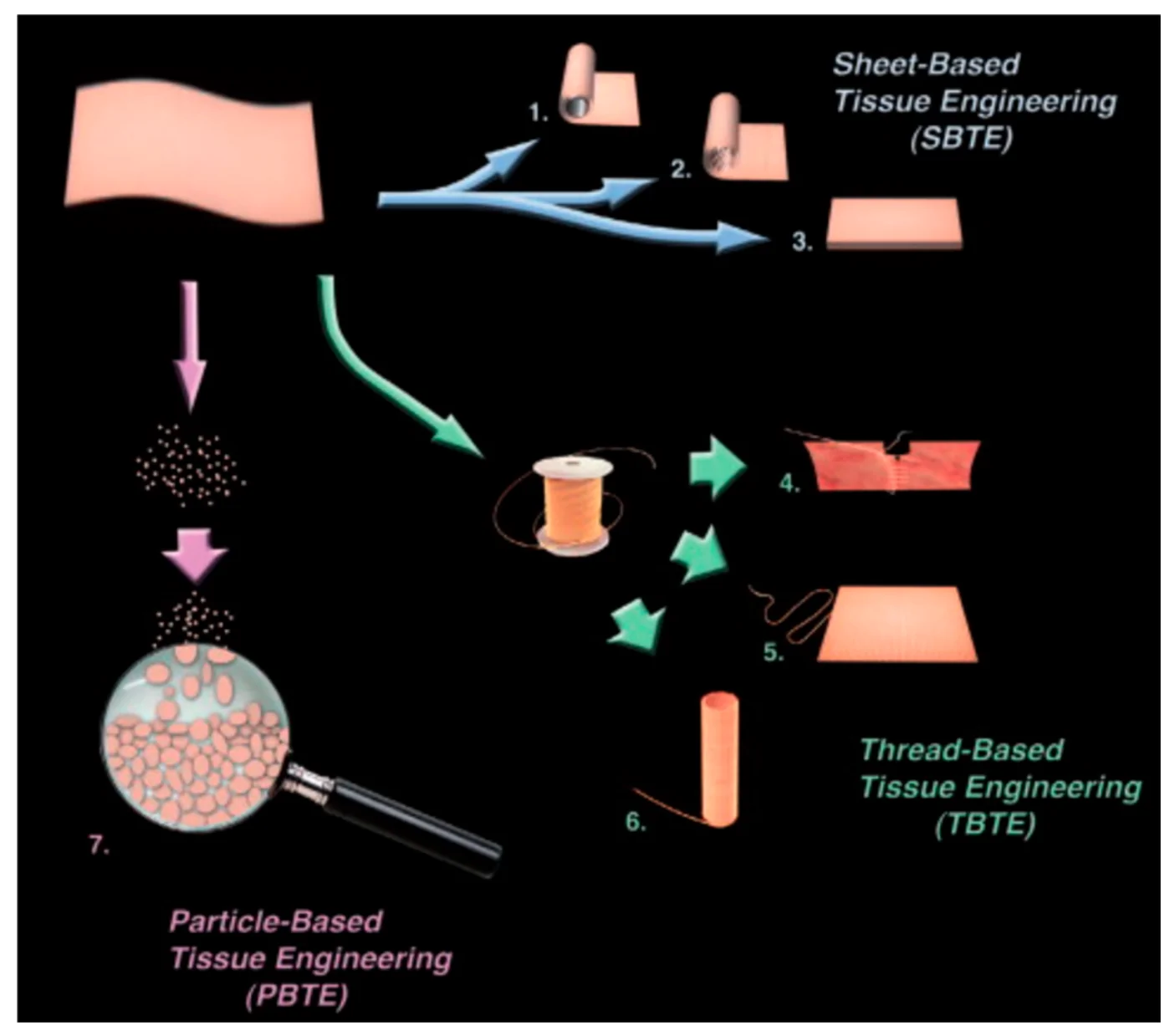
Schematic diagram of tissue engineering by self-assembly. (Peck, M. et al.) [26].

To address the scalability limitations of individual spheroids, where metabolic cores typically fail beyond 400–600 μm, scientists are pivoting toward tissue engineering by self-assembly (TESA) and advanced bioprinting to construct larger, integrated architectures [2,25]. One prominent solution involves the fusion of multicellular spheroids and cylinders via 3D bioprinting to create macroscale structures, including branched vascular conduits (Figure 5) [2].

For instance, research by Forgacs and colleagues demonstrated that precisely depositing printed microtissue building blocks into a mold allowed them to fuse into confluent tubes which, after 21 days of bioreactor maturation, achieved burst pressures of 773 mmHg [2]. While this “bottom-up” assembly creates high-density cell constructs without the potential for scaffold-induced inflammation, the challenge remains to provide an internal perfusion network that mimics the natural vascular hierarchy to support long-term survival in constructs exceeding several millimeters (Table 1) [20,25].

### 2.2. Maintaining Function

#### 2.2.1. Maintaining Patency in TEVG

The patency of vascular constructs is uniquely dependent on the maintenance of a functional, non-thrombogenic endothelial lining [1,5]. Endothelial cells are highly sensitive to the hypoxia induced in a synthetic SDVG. In the absence of a healthy, confluent endothelial layer, synthetic surfaces are directly exposed to blood, leading to the absorption of plasma proteins and blood cells that activate coagulation cascades [1]. This process results in the aggregation of insoluble fibrin and platelets, causing acute thrombosis [1]. Simultaneously, intimal hyperplasia (IH) is triggered as smooth muscle cells (SMCs) migrate from surgical connection sites toward the inner lining of the graft [1]. These cells transform from a quiescent state into a dedifferentiated, proliferative type, narrowing the vessel lumen and further slowing blood flow, which creates a dangerous feedback loop that promotes more clotting [1,4].

This is particularly critical in small-diameter vascular grafts (SDVGs) (≤4 mm), where decreased blood flow velocity exacerbates protein adsorption and lumen occlusion [1,4]. If a graft material is merely “biotolerated” rather than truly biocompatible, the body isolates it within a non-vascularized fibrotic capsule, which acts as a barrier to nutrient exchange and biological integration [4], increasing the IH in the body.

The eventual process of inosculation (the functional unification of donor and host networks) with SDVGs typically takes days to weeks, during which construct viability often declines [16]. To bypass this “lethal lag”, researchers are developing hierarchically organized constructs that incorporate suturable macrovessels, allowing for immediate surgical anastomosis and perfusion upon transplantation [16].

#### 2.2.2. Physiological Maturation and In Vivo Validation Across Species

Achieving functional integration requires that engineered constructs mimic the mechanical and electrochemical properties of native tissue. Bioreactor conditioning, specifically pulsatile flow, is critical for providing the hemodynamic cues necessary to organize smooth muscle cells (SMCs) into mature, contractile structures [2,27]. Niklason’s group achieved vessel burst pressures of 2150 mmHg (exceeding that of the human saphenous vein) by culturing cell-seeded scaffolds under pulsatile flow for 8 weeks [2,27].

#### 2.2.3. Immunogenicity Control of TEVG

TEVG offers a promising alternative to synthetic conduits by supporting host-driven vascular regeneration and growth. However, immunogenicity remains a major barrier to long-term function and clinical translation. Immune responses can arise from multiple sources, including residual donor cellular components, xenogeneic extracellular matrix proteins, degradation byproducts of scaffold materials, and implanted or delivered cells. These stimuli activate innate immune pathways, particularly macrophages and foreign body responses, leading to chronic inflammation, fibrosis, calcification, neointimal hyperplasia, and graft stenosis or failure. While complete immune suppression is undesirable because controlled inflammation is essential for constructive remodeling, excessive or prolonged immune activation disrupts tissue regeneration and vascular maturation.

To address immunogenicity, CRISPR/Cas9 genome editing is used to disrupt HLA genes in iPSCs, creating hypoimmunogenic “universal” donor cells [28,29]. To overcome the formidable immune barriers of allogeneic transplantation, where the host’s recognition of foreign MHC and HLA leads to graft rejection, researchers are utilizing CRISPR/Cas9 genome editing to engineer hypoimmunogenic induced pluripotent stem cells (iPSCs) [27,29]. This state-of-the-art strategy circumvents the prohibitive costs and delays of autologous cell production by creating “universal” donor tissues [2,29]. By disrupting specific HLA genes to hide donor cells from host T cells, scientists have generated iPSC lines estimated to be compatible with over 90% of the world’s population [28,29]. Beyond gene deletion, researchers “camouflage” these cells by overexpressing protective markers like CD47, a signal that prevents destruction by the innate immune system, and inducing PD-L1 to suppress local immune responses [29]. This technology is particularly vital for highly immunogenic vascular components like endothelial cells, providing a more definitive solution than decellularized scaffolds, which can still elicit chronic inflammation [2,27]. While specialized cases like pediatric vascular reconstruction can leverage neonatal immune tolerance for parent-derived grafts, CRISPR-mediated editing offers a scalable, “off-the-shelf” solution for the broader adult population by producing living tissues that host immune systems simply do not recognize as foreign [29,30]. Furthermore, clinical experience with parent-derived allogenic vein grafts in infants suggests that neonatal immune tolerance, due to immunologic immaturity, can allow for successful graft acceptance even with genetic differences, offering a unique solution for pediatric vascular reconstruction [30]. These multifaceted strategies address the diffusion limit, clinical delivery, scaling, and immunological barriers essential for the translation of personalized tissue engineering [2,29].

## 3. Section 2: Challenges of Microvasculature Development

### 3.1. Fabrication Strategies: Methods and Limitations

#### 3.1.1. Direct Bioprinting of Microvasculature

**Figure 6:**
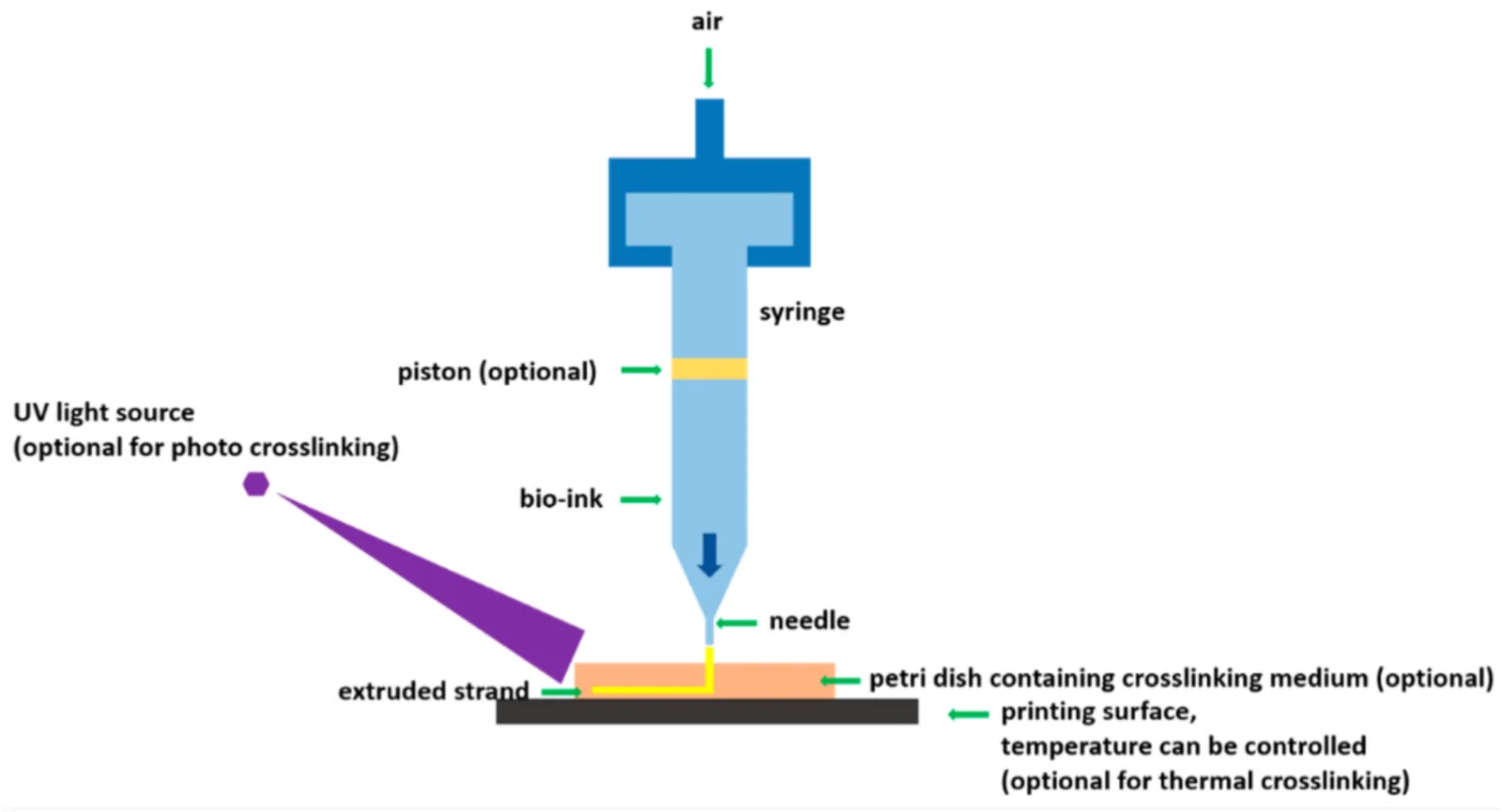
Schematic of extrusion based-bioprinting. (You et al., 2017) [33].

Bioprinting enables the precise spatial positioning of living cells and biomaterials to recreate complex anatomical architectures [31]. Extrusion-based bioprinting [32] typically employs a nozzle-based system to dispense cell-laden hydrogels according to a computer-aided design (CAD) model (Figure 6).

Extrusion-based 3D bioprinting faces several critical technical and biological trade-offs when applied to microvasculature creation [10,31]. The technique is primarily constrained by a critical trade-off where achieving high cell viability often comes at the cost of reduced printability [23], as nozzle-based extrusion processes subject encapsulated cells to damaging shear stresses and high dispensing pressures that compromise cell viability and negatively alter cellular phenotypes [10,23,31]. Compounding this, the resolution of extruded cell-laden structures is limited to approximately 50 μm, making it challenging to replicate the fine, hierarchical architectures of native capillary beds [10,31]. These constructs further exhibit poor mechanical integrity at the interfaces between printed lines, creating structural vulnerabilities that may lead to failure under physiological loads [31], and sacrificial bioprinting methods remain susceptible to operational risks such as clogging and bursting when fabricating intricate small-diameter branches [31]. To overcome these hurdles, researchers are exploring alternative methods like microscale continuous optical bioprinting (μCOB), which utilizes digital light processing (DLP) to eliminate nozzle-related shear stress and produces constructs without artificial interfaces for improved structural stability [31].

#### 3.1.2. Indirect Bioprinting of Macrovasculature (Use of Sacrificial Materials)

**Figure 7:**
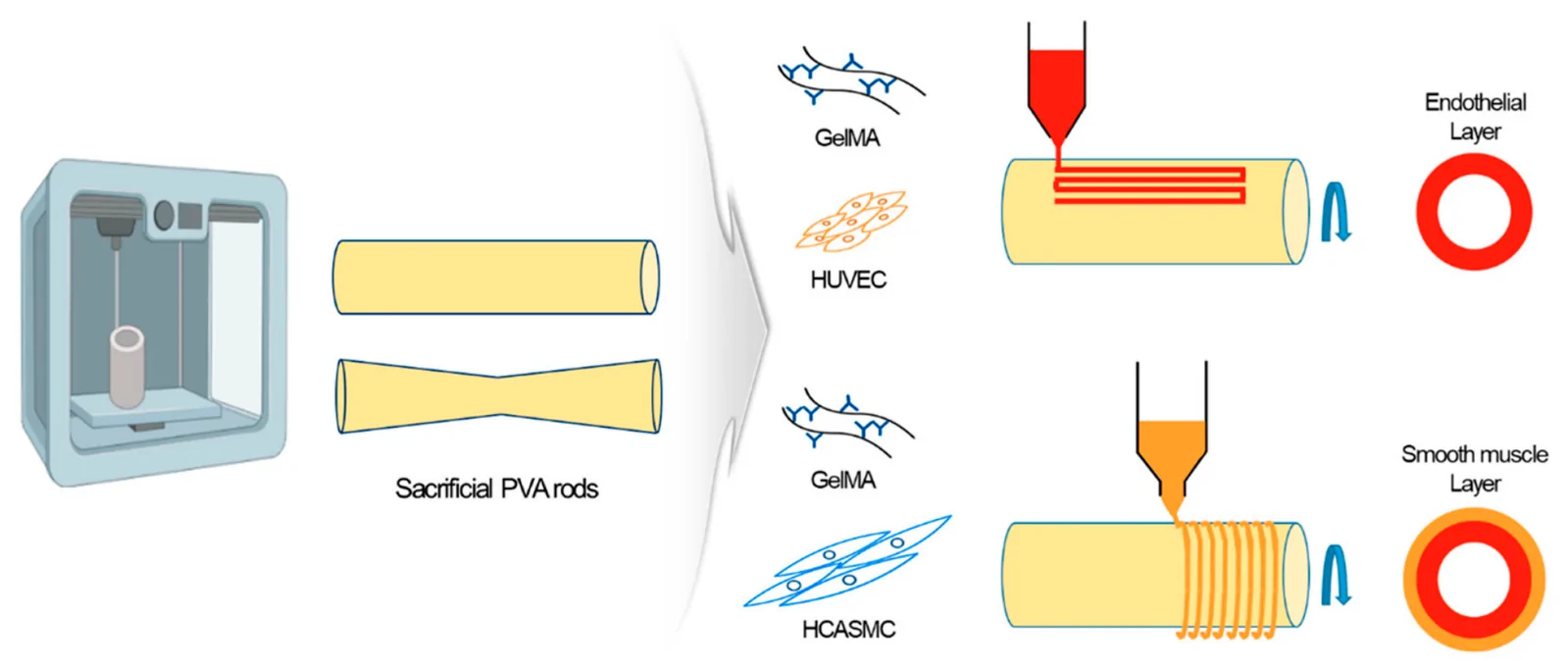
Schematic of sacrificial bioprinting (Lee J et al., 2024) [34].

This involves printing a sacrificial material (e.g., carbohydrate glass or specialized gelatin) to form a temporary template for vascular channels. Once the surrounding tissue-laden hydrogel is cross-linked, the sacrificial ink is dissolved, leaving behind a perfusable microvascular network that can be seeded with endothelial cells (Figure 7, Table 2) [22,31]. 

These bioprinted conduits allow for the immediate transport of nutrients and oxygen, bypassing the lethal lag associated with spontaneous angiogenic ingrowth from host vessels. However, a key challenge involves the complex extra process of dissolving sacrificial materials and subsequently perfusing endothelial cells into the resulting microchannels [31]. This perfusion stage carries significant operational risks, as the delicate networks are susceptible to clogging and bursting, particularly when trying to engineer large-scale tissues with small vessel branches [31]. The requirement to remove filler materials, such as bioprinted agarose rods, often imposes strict limits on the scalability and geometric complexity of the final construct [23]. Furthermore, sacrificial inks like carbohydrate glass can produce rigid, sharp-edged lattices that fail to replicate the smooth, curved morphology of native human vasculature [35]. Beyond initial fabrication, achieving long-term vascular stability and functional branching remains a persistent challenge for thicker, cell-dense cardiac tissues [10].

### 3.2. Maintaining Function

#### 3.2.1. Maintaining Viability in Microtissue

For microtissue, viability remains a key challenge because sufficient oxygen levels can only diffuse ~200 μm from blood vessels in cell-dense tissues [16]. When engineered constructs exceed this physical threshold, the resulting diffusion limits of oxygen and nutrients mean that the transport of essential molecules becomes insufficient for the desired cell behavior [36]. Consequently, in larger or highly metabolic constructs, a lack of vascularization and oxygen in the center leads to ischemia followed by necrosis within the first week after implantation [36]. This formation of a central necrotic core is a critical failure point that is often further accelerated because carrying cells within scaffolds further increases local metabolic demand and exacerbates the oxygen deficiency challenge [37]. To address these constraints, researchers must develop artificial environments that promote rapid host tissue integration and vascularization upon implantation to prevent ischemic cell death [36].

Assessment of viability involves a multi-modal approach, including simple metabolic assays like MTS/MTT and live/dead staining [5,21,36]. However, functional performance is increasingly used as the gold standard for success, including measuring contractile force generation, conduction velocity (CV), and longitudinal monitoring of calcium transients (GCaMP6) in live models [38,39]. Advanced imaging techniques, such as laser Doppler perfusion imaging and micro-CT perfusion, provide real-time data on blood flow and the functional connectivity of newly formed hybrid donor–host vascular networks [36,39]. Monitoring macrophage polarization toward a pro-healing M2 state also provides critical insights into whether a construct is achieving true biological integration [4].

Prevascularization is the primary strategy used to overcome the 200 μm oxygen diffusion limit [16,36]. This involves establishing microvascular networks in vitro using ECs alongside stabilizing mural cells, such as pericytes or mesenchymal stem cells (MSCs) [19,40,41]. Engineered microvessels formed solely by endothelial cells (ECs) represent an “incipient” or “primitive” state that is highly unstable. Without the recruitment and stabilization provided by perivascular mural cells, such as pericytes or smooth muscle cells (SMCs), these nascent capillaries are prone to regression and failure. Co-culture systems must therefore balance the complex, reciprocal paracrine signaling between ECs and these supportive populations to ensure long-term vascular integrity.

Current research is increasingly focused on combining top-down engineering (bioprinting large channels) with bottom-up self-assembly (microvascular sprouting) to circumvent the core necrosis seen in large spheroids and organoids [25,42].

A pioneering study by Zhang et al. [25] utilized clickable poly(ethylene glycol)-norbornene (PEGNB) microgels as a versatile suspension bath to support the bioprinting of mesoscale channels (600–1000 μm) using sacrificial bioinks. In this model, endothelial cells (ECs) and stromal cells co-embedded within the microgel slurry prior to printing were able to self-assemble into mature microvascular networks within the interstitial voids of the construct over 7 days [25]. Crucially, the interstitial porosity of these granular hydrogels facilitated mass transport of nutrients and oxygen, supporting the survival of metabolically active cells throughout volumes exceeding 500 mm^3^, thereby effectively pushing past the diffusion barrier that limits standard spheroids [25].

These microgels support suspension bioprinting using an Allevi 3 bioprinter to embed sacrificial bioinks (such as gelatin or Pluronic F-127) within a jammed microbead slurry [25]. Once the bioink is evacuated, it leaves a patterned void space (600–1000 μm in diameter) that can be perfused with media or cells, effectively providing a “top-down” engineered channel for advective transport, while the interstitial voids between the clicked microbeads support “bottom-up” microvascular self-assembly for diffusive exchange [25].

#### 3.2.2. Enhancing Organoid Maturation Through Perivascular Co-Culture and Mechanical Cues

To overcome the lack of functional vascular compartments in hiPSC-derived organoids, scientists are implementing tri-culture systems and mechanical conditioning to drive physiological relevance [25,29]. Embedding ECs alongside supportive stromal populations, such as normal human lung fibroblasts (LFs) or mesenchymal stem cells (MSCs), has been shown to support microvascular morphogenesis in a pericyte-like manner [25,42]. These stromal cells localize to the vascular structures, expressing markers such as *α*-SMA, PDGFR-*β*, and NG2, which stabilize the developing lumens and promote the secretion of basement membrane proteins like laminin *β*-1 and collagen IV [25,42]. Furthermore, the application of mechanical loading and cyclic stretch within bioreactors is being used to enhance the maturation of these vascularized constructs, as seen in co-cultured human ESC-derived cardiac constructs where mechanical stress significantly boosted cardiomyocyte proliferation and vessel-like formation [2,42].

#### 3.2.3. In Vivo Chamber Models for Spheroid Survival and Functional Integration

For large-scale spheroid-based grafts that require immediate and robust perfusion, in vivo chamber models offer a mechanism to leverage the host’s regenerative environment to establish a blood supply [42]. By implanting microtissue aggregates or spheroids into a protected space around a surgically created arteriovenous (AV) loop, researchers can utilize the rapid vascular sprouting from the loop to integrate with the implanted donor material [42]. Yap et al. [43] demonstrated that this approach significantly enhanced the survival and differentiation of liver progenitor cell spheroids in a vascularized rat chamber, creating a supportive niche that bypassed the diffusion limitations inherent to in vitro culture [42]. This strategy allows for the engineering of compact contractile or metabolic tissues up to 0.5 mL in volume, with the vascular pedicle subsequently enabling microvascular transplantation of the matured organoid to the definitive injury site [2,42].

#### 3.2.4. Fiber-Guided Vascularization

Fiber-guided approaches utilize biodegradable electrospun microfibers to provide contact guidance, directing endothelial cell (EC) organization into organized, vessel-like networks [35]. These hybrid scaffolds can achieve functional anastomosis with host vasculature in as early as 1 day post-implantation, significantly faster than traditional angiogenic ingrowth [35]. Hierarchical vascularization has also been achieved by machining macropores (~150 μm) into electrospun PCL macrovessels, allowing ECs to migrate from the lumen into surrounding cell-laden hydrogels through sprouting angiogenesis [16]. While these methods enhance structural maturation and directional growth, they often struggle to replicate the complex branching hierarchy of native vascular trees [16].

## 4. Section 3: Future Considerations and Measures of Success

The future positioning of vascular engineering is characterized by a definitive shift from the manufacture of static, bioinert conduits toward bioinstructive platforms that leverage the host organism as an active bioreactor. A primary technological frontier is the integration of intraoperative bioprinting (IOB) with innovative surgical maneuvers such as micropuncture (MP) [44]. This synergy utilizes an ultrafine 60 µm microneedle to create targeted perforations in the host macrovasculature, inducing rapid microvascular formation and anastomosis within a 10-day post-operative window [44]. This represents a significant advancement over earlier microsurgical techniques, such as arteriovenous loops (AVL), which often required several months for functional vascularization [42,44]. Simultaneously, technologies are evolving toward scaffold-guided in vivo self-assembly, as seen in bioengineered tubular vascular constructs (BTCs) [20]. By implanting 3D-printed biodegradable polylactic acid (PLA) scaffolds subcutaneously, the host’s endogenous cells and extracellular matrix (ECM) are recruited to form functional autologous tissues within a 14-day cycle, aligning fabrication with the clinical window for elective surgeries [20].

Furthermore, future systems are positioned to address the hierarchical complexity of vascular networks through multiscale design strategies. The use of clickable poly(ethylene glycol)-norbornene (PEGNB) microgels allows for the suspension bioprinting of mesoscale channels (50–1000 µm) while supporting capillary-scale self-assembly within the interstitial voids of the granular matrix [25]. This dual-approach addresses both advective flow requirements and diffusive transmural transport [25]. To combat the “vicious cycle” of thrombosis and intimal hyperplasia common in small-diameter vascular grafts (SDVGs), future technologies are also integrating dual-gas release systems—specifically nitric oxide (NO) and hydrogen sulfide (H2S)—into zwitterionic coatings to selectively promote endothelialization while inhibiting smooth muscle cell proliferation [45].

### 4.1. Considerations for Future Technologies and the Utility of Optimized Cell Ratios

Future engineering efforts must prioritize clinical timeliness and mechanistic biological control to overcome the limitations of current translational models. A critical consideration identified in recent research is the optimization of cellular ratios, specifically regarding the co-culture of endothelial cells (ECs) and stromal cells. While traditional co-cultures with fibroblasts have been standard for 2D tubular formation, evidence now suggests that in 3D bioprinted environments, an endothelial-cell-only strategy may be more effective for inducing rapid, continuous vascular networks [44]. This is attributed to the observation that stromal cells like fibroblasts may only be transiently required during early morphogenesis and their continued high presence can actually lead to discontinuous structures [25,44]. This knowledge is immensely useful, as it simplifies regulatory hurdles for cell-laden constructs and streamlines intraoperative fabrication protocols [44].

Another vital consideration for next-generation grafts is the mitigation of pathological fibrosis, which often serves as a barrier to nutrient transfer and capillary ingrowth [46]. The discovery of the APOE-mediated profibrotic pathway highlights that apolipoprotein E expression increases significantly during vascular regeneration, recruiting profibrotic macrophages that secrete IGF-1 and drive excessive ECM deposition [46]. Future technologies may require the adventitial delivery of gene therapies, such as AAV-shRNA, to knock down APOE and maintain graft compliance [46]. Additionally, the move toward granular hydrogels provides a necessary physical microenvironment; the interconnected microporosity of these materials facilitates cell migration and nutrient communication more effectively than bulk hydrogels [25,44].

### 4.2. Lessons from Successful Graft Types

Vascular grafts have historically faced higher failure rates than other engineered tissues, but important lessons can be derived from areas of relative success. One primary lesson is the limitation of diffusion. Engineered tissues such as skin and bladder have successfully translated to the clinic because they are thin or avascular, allowing metabolic needs to be met via simple diffusion (<200 µm) [42]. For vascular grafts, this underscores the necessity of engineering the hierarchical transport system itself rather than just a structural conduit [2,25]. Furthermore, the success of limb-sparing surgeries in infants using parental saphenous vein allografts demonstrates the potential of neonatal immune tolerance [30]. This suggests that leveraging unique immune windows could alleviate the need for aggressive immunosuppression in specific pediatric populations [30].

Comparative analyses also reveal that functional passiveness is superior to total bioinertness. While synthetic grafts like ePTFE or Dacron are standard for large vessels, they fail in small diameters because their surfaces do not actively prevent the “vicious cycle” of thrombosis and intimal hyperplasia [27,47]. Successful modern strategies are learning to create actively antithrombogenic “living” surfaces using zwitterionic interfaces and bioactive gas release [45,47]. These “living” grafts mimic native arterial structure more closely than simple inert coatings, providing a blueprint for long-term patency [45].

### 4.3. Critical Conclusions and Integrative Directions

The ultimate solution for vascular repair lies in the synergy of the “tissue engineering triad”: engineered scaffolds, living cells, and controlled growth factor delivery systems [44,48]. A major barrier remains our incomplete knowledge of cell fate post-implantation; research indicates that cell retention can be as low as 5% after 24–48 h in human subjects [49]. Therefore, future clinical progress is contingent upon the integration of molecular imaging (e.g., microPET or bioluminescence) to track cell survival and correlate it with functional outcomes [49].

Furthermore, the polarization of macrophages is a decisive determinant of graft success. Promoting an early M2-type macrophage phenotype is essential for reducing inflammation and facilitating the transition of smooth muscle cells from a synthetic to a contractile state, thereby preventing vascular stenosis [45,46]. Finally, the mechanical property of compliance matching remains a primary driver of intimal hyperplasia [27,47]. Future designs must prioritize emulating the native “J-shaped” stress–strain response [2], ensuring the graft expands and contracts harmoniously with the host vessel to prevent abnormal shear stresses [20,46]. These integrated findings suggest a future where personalized vascular constructs function as dynamic, autonomous biological systems rather than as mere prosthetic devices.

## Figures and Tables

**Table 1:** Principles, advantages, limitations, strategies to improve and key challenges for scaffold-based vs. spheroid-based grafts.

Aspect	Scaffold-Based TEVGs	Spheroid-/Self-Assembly-Based TEVGs
**Fabrication principle**	Cells are seeded onto 3D biomaterial scaffolds, commonly fabricated from synthetic polymers such as PCL or PLGA.	Scaffold-free multicellular spheroids are formed and subsequently fused or bioprinted into larger vascular structures.
**Main advantages**	Provides strong mechanical support, tunable properties, ECM-like fibrous architecture, and potential control over pore size and graft geometry.	Promotes high cell–cell and cell–ECM interactions, natural tissue organization, and avoids scaffold-associated foreign body reactions.
**Major limitations**	Poor cell infiltration, foreign body response and fibrotic encapsulation, compliance mismatch, and possible intimal hyperplasia.	Limited by oxygen and nutrient diffusion; spheroids above ~400–600 μm may develop necrotic cores, making scale-up difficult.
**Strategies to improve performance**	Electrospinning combined with macropore formation, microneedle stamping, porogen leaching, and mechanical/compliance optimization.	3D bioprinting, tissue engineering by self-assembly (TESA), spheroid fusion, and incorporation of pre-vascularized units.
**Key challenge for clinical translation**	Achieving sufficient porosity and host integration while maintaining mechanical strength and vascular compliance.	Creating a stable hierarchical perfusion network that supports long-term survival and maturation of macroscale grafts.

**Table 2:** Comparison of 3D bioprinting techniques: direct bioprinting vs. sacrificial bioprinting.

Aspect	Direct Bioprinting of Microvasculature	Indirect/Sacrificial Bioprinting of Macrovasculature
**Method**	Cell-laden hydrogels are directly deposited through a nozzle following a CAD model.	Sacrificial material is printed, embedded in hydrogel, then removed to create hollow vascular channels.
**Main advantage**	Precise spatial placement of cells and biomaterials.	Creates immediately perfusable channels for oxygen and nutrient transport.
**Main limitation**	Shear stress and high pressure can reduce cell viability and alter cell phenotype.	Requires extra steps for sacrificial material removal and endothelial cell perfusion.
**Resolution/structure**	Limited to ~50 μm resolution, making native capillary networks difficult to reproduce.	Better suited to larger vessels, but small branches may clog, burst, or be geometrically limited.
**Key challenge**	Balancing printability, cell viability, resolution, and mechanical stability.	Achieving scalable, complex, stable, and smoothly branched vascular networks.

## Data Availability

No new data were created or analyzed in this study. Data sharing is not applicable to this article.
